# Self-Reported Violence Experienced by Swiss Prehospital Emergency Care Providers

**DOI:** 10.1155/2021/9966950

**Published:** 2021-12-17

**Authors:** Simon Savoy, Pierre-Nicolas Carron, Nathalie Romain-Glassey, Nicolas Beysard

**Affiliations:** ^1^Emergency Department, Lausanne University Hospital, Lausanne, Switzerland; ^2^Violence Medical Unit, University Centre of Legal Medicine, Lausanne University Hospital, Lausanne, Switzerland

## Abstract

**Background:**

Workplace violence is a serious and increasing problem in health care. Nevertheless, only few studies were carried out concerning this topic and then mainly in English-speaking countries. The objectives were to describe the acts of violence experienced by prehospital emergency care providers (PECPs) in the western part of Switzerland between January and December 2016 and to assess the consequences for subsequent PECPs behaviors.

**Methods:**

An observational cross-sectional study, carried out using an online survey, has been sent to all 416 PECPs in the Canton of Vaud, in the western, French-speaking, part of Switzerland. The survey contained items of demographic data and items to assess the type and consequence of violence sustained. This was classified as five types: verbal assault, intimidation, physical assault, sexual harassment, and sexual assault.

**Results:**

273 (65.6%) PECPs participated in the survey. During 2016, workplace violence was reported by 229 survey participants (83.9%). Most declared to be the victim of such violence between one and three times during the year. In all cases of violence described, the patient and/or a relative initiated aggressive behavior in 96% of cases. Verbal assaults were the most common (99.2% of all acts), followed by intimidation (72.8%), physical assault (69.6%), and sexual harassment (16.3%). Concerning physical assault, PECPs were predominantly victims of spitting and/or jostling (50%). After a violent event, in 50% of cases, the PECPs modified their behavior owing to the experience of workplace violence; 82% now wear protective vests, and 16% carry weapons for self-defense, such as pepper sprays. Seventy-five percent changed their intervention strategies, acting more carefully and using verbal de-escalation techniques or physical restraints for violent patients.

**Conclusions:**

Workplace violence is frequent and has significant consequences for PECPs. In order to increase their own security, they increased their protection. These results illustrate their feelings of insecurity, which may have deleterious effects on work satisfaction and motivation. *Trial Registration*. Our article does not report the results of a health care intervention on human participants.

## 1. Background

Violence encountered at work is a serious problem particularly affecting healthcare workers who are considered to be at high risk [1–3]. Such violence is primarily verbal abuse [1,4–22] and is committed by patients and their entourage [1,9,13,15,17–21,23–27]. Inappropriate behavior is often the result of intoxication by alcohol or psychostimulants, a psychiatric disorder, or both [1,15,17,19,21,24–26,28]. Emergency and psychiatric services are consequently regularly confronted with this problem [1,23].

Likewise, PECPs are extremely exposed to such violence, being met by recurrent and regular abuse in approximately 1–5% of all interventions concerning agitated or violent patients [26,28,29]. Studies suggest that, globally, PECPs are faced with violence in 4-5% of their call-outs [1,11,26] and 80% will have endured some kind of such abuse in the lifetime of their career [1].

The high risk of violence is a major professional stress factor [11] causing some PECPs to reduce their professional activity, or to start to wear protective jackets or to carry self-defense tools [1,28,30], such as pepper sprays, stun guns, and, notably in the United States of America, firearms [18].

Although the nature of this abuse is known and that studies from many countries have been published, no recent data exist from Switzerland on this subject.

Thus, this study gathered information concerning PECPs in the Canton of Vaud enduring violence in their professional capacity during the year 2016.

## 2. Methods

### 2.1. Study Setting and Population

This is a cross-sectional study carried out by use of an online survey. It sets out to describe violence sustained by PECPs in the Canton of Vaud during their call of duty between January 1^st^ and December 31^st^ 2016. The Canton of Vaud is located in the western French-speaking part of Switzerland and has about 780,000 people. Emergency medical services (EMS) consist of paramedics and emergency medical technicians; depending on the situation, an emergency physician may be dispatched by road (Service Mobile d'Urgence et de Réanimation (SMUR)) or by air (helicopter emergency medical service (HEMS)) in the event of a life-threatening emergency or in support of paramedics, at their request.

### 2.2. Study Protocol

For the survey, a detailed definition of workplace violence was used, inspired by the Canadian Centre for Occupational Health and Safety [31], refined for use by PECPs according to the literature available in 2016. Workplace violence was thus defined as *“any act at work during which a person is abused, menaced, intimidated, physically assaulted, sexually harassed or assaulted”* (Table 1). A preliminary survey was tested confidentially by four trained paramedics and two emergency physicians during the autumn of 2016. The aim was to gauge impressions concerning the clarity and pertinence of the questions posed. Remarks and comments given served to edit and modify the final version.

The survey (Annex 1) contains seven questions on participants' demographic data and four sections with 30 questions detailing abuse encountered, its consequences, and changes in resulting practice for PECPs. Most questions are closed (yes/no answers) or have multiple answer choices. The final section has three open-ended questions. At the end, a free space allows comments. After data collection and in order to facilitate the analysis, answers were categorized according to similarity. Answers related to reaction to a violent patient were divided into three distinct groups: safety reactions (subdivided into two groups), de-escalation, and restraining activity. A detailed explanation of the study purpose, together with details on how to respond online, was sent to all EMS chiefs in the Canton of Vaud, as well as emergency physician, active during the year 2016. People were asked to forward the questionnaire to all their staff and indicate how many were thus sent to. Such participation implied primary consent.

### 2.3. Data Handling and Analysis

Data were anonymously collected through the online survey Google Forms^©^, a collaborative software develop by Google LLC (Mountain View, California, US), allowing direct encryption of data and its export for analysis, using advanced encryption algorithm AES 128 bits or more [32]. Since the study involved volunteer PECPs and did not include patients, the ethics committee on human research of the Canton of Vaud, Switzerland, gave a no objection agreement (CER-VD decision in June 2016).

## 3. Results

The survey was distributed to all active PECPs (416 in 2016) working in the Canton of Vaud. Two hundred seventy-three (65.6%) responded. Of these, 72.2% were nursing homes and 38.3% were emergency physicians.

Among the study population, 78 women (28.6%) and 195 men (71.4%) answered the survey. This proportion observed between men and women corresponds to the gender distribution of paramedics: 30% women and 70% men [28]. Three age groups were considered: “under 35 years of age,” “35–49 years of age,” and “50 years of age and over.” The “under 35” and “35–49″ age groups were the most represented, with 43.6% and 43.2% of participants, respectively. The most represented profession is paramedic. It corresponds to 157 participants (57.5%), while ambulance technicians represent 28.2% and physicians represent 11.4% of the participants. Ambulance students at the end of their training, employed in ambulance services during the survey, were also invited to answer the survey. They represent 2.9% of the study population. The complete demographic characteristics are detailed in Table 2.

Of the 273 PECPs, 229 (83.9%) stated they had been the subjects of abuse at work during 2016 and 257 (94.1%) at least once during their career. Verbal aggression was commonest (99.2%) (Table 3), followed by intimidation, physical assault, and finally sexual harassment, which was more frequent amongst females (34.2%). Sexual assault was the least frequent of all (1.2%).

During year 2016, a majority of PECPs who encountered violence at work had been confronted between one and three times a year, which also applies to the frequency of violence during the career.

These acts of violence occurred in more than two thirds of cases (69%), in the evening or night periods. A majority took place on-site (56.4%), whether in a public place or at home, and 26.3% inside the ambulance. In 16.6% of cases, the assault on PECPs started or continued upon arrival at the hospital. In 92.7% of cases, the patient was the aggressor. His family or friend was involved in 57% of events. In rare cases (2.2%) a witness or bystander was involved. In ten cases (3.7%), a colleague was the perpetrator.

Almost all of the participants who reported assaults described verbal abuse (99.2%). The other types of violence reported are described in Tables 3 and 4. Concerning intimidation, PECPs were essentially victims of menacing or frightening behavior (53.1%). Other types of intimidation were much less frequent: 14 (5.1%) were chased physically by an aggressor and 13 (4.8%) were verbally abused at home or in private.

Types of physical aggression varied (spitting, pushing, kicking, punching, stabbing, etc. (Table 4)) of which spitting and pushing were commonest, but 17 (6.2%) were assaulted with a knife or a sharp object.

Both men and women have been victims of sexual harassment. Women were however more often affected (34.2% against 9.2%). Most of the cases (78%) were jokes and humiliating or offensive remarks of a sexual nature. A sexual assault was encountered by three trained paramedics, two women and a man, involving petting and touching of the genitals, breasts, or buttocks. No unwanted sexual acts have been reported. All shades of violence had a psychological impact; minor consequences were reported from verbal abuse (43.9%), intimidation (47.6%), sexual harassment (21.4%), and sexual abuse (33.3%). More major consequences resulted from physical aggression (Table 5). Thus, 51.4% of PECPs suffered from psychological consequences following a physical aggression and 14% of them were moderate. Furthermore, numerous PECPs indicated being afraid at their workplace, with 221 respondents experiencing fear at least once during a rescue. Sixty-four percent responded that they were rarely afraid (<25% of cases), 40 (15%) expressed occasional fear, and 30 (11%) were afraid to go to work. Finally, just over half (54%) considered violence to be part of their job. There were 135 PECPs (49.5%) who indicated that they had modified their work practice as a result of experiencing violence. The most frequent change was the wearing of a protective vest (82%), followed by the wearing of gloves, long-sleeved shirts or coats, protective glasses, and masks. Another change for 21 PECPs (15.6%) was the carrying of weapons (mainly pepper spray or a knife) or having one available in the ambulance. As a result of experiencing violence, PECPs took several different measures (Table 6); more than half (59.3%) called for help, mainly the police. In addition, most participants (83.2%) felt well supported by the police.

## 4. Discussion

This study shows that workplace violence, reported by 229 PECPs (83.9%) in 2016, is common in prehospital emergency care. This violence is mainly verbal assault made by patients. Although verbal, they nevertheless have psychological repercussions on PECPs. In Switzerland, PECPs are, as their colleagues elsewhere, exposed to violence at work. A Canadian study showed that 75% of PECPs were exposed to violence during the period of one year [11]. Similarly, verbal abuse was the most common form of violence, as most recent studies report [1,4–8,25,27]. However, some differences are noted regarding other studies, notably from the USA, where violence was more severe. In a study in prehospital emergency services in New England, 80% of participants were physically assaulted and 40% required hospital treatment [10]. Although in this study, physical assault was relatively common (present in 70% of violent events), it never resulted in permanent injury and never was deadly unlike a few cases in the United States [33].

Nevertheless, psychological repercussions occurred in about 50% of cases, although they were mostly minor: minor anxiety, poor memory of the incident, mistrust, or the onset of tobacco use. Although defined as minor effects [34], these repercussions nevertheless have a long-term impact, as shown in a Spanish study which demonstrated a greater risk of emotional fatigue or burnout [35]. In addition, 81% of participants were afraid to be on the scene. This must have a long-term negative effect on job satisfaction and motivation [1,36–39]. Demotivation as well as fear at work can have a detrimental effect on medical care provided by PECPs. Thus, the psychological repercussions should not be considered negligible and must be taken into account. The number of PECPs who had started wearing protective vests, and carrying special equipment, is not negligible. The decision to carry a weapon (even if it is not a gun but only a pepper spray) has legal implications and could make PECPs look like policemen. This appearance could have the opposite effect to that sought by generating more violence, the paramedics being taken for police. Finally, three participants (1.1%) indicated they would seek to resign because of the violence they suffered. Because our survey did not specifically address the desire to change careers due to violence encountered at work, it is not possible to establish how often it occurs. However, it was found that violence in the workplace was a major factor in nurses' desire to change jobs [38].

### 4.1. Limitations and Strengths

Although with 65.6% participation this study can be considered representative, our results may be overestimated. Indeed, if the PECPs did not consider the subject important enough or if they had not themselves been exposed to violence, it is possible that they did not answer the survey. It seems that emergency physicians are less concerned than PECPs as only 38.3% responded, in comparison to PECPs (72.2%). This may be because they themselves were much less exposed to violence but also because no medical union strongly encouraged participation. Our survey did not have a mechanism preventing a participant from answering multiple times. However, we analyzed the personal data (year of birth, function, percentage, and number of years of practice) to rule out possible duplicates. We did not identify such duplicates so we supposed nobody answered more than once.

Interpretation of consequences of violence must be made with caution as the choice of answers was predefined and was not categorized by a specialist; thus, the precise risk of secondary anxiety or posttraumatic stress cannot be assessed from this population. In addition, the questionnaire was pretested only by a small group of people. Although their responses could not be extrapolated collectively, to other regions of Switzerland or Europe, recent studies [4–7,36,37,40–42] show that workplace violence is a transversal phenomenon and that there are many similarities in the violence against healthcare providers worldwide. However, the high overall response rate, the quality of replies, and the analysis of qualitative data (by grouping of open questions, and of those with similar categorization) are strengths of this study, which remains the only one of its type to be conducted in Switzerland and one of the first in Europe.

## 5. Conclusion

This study shows that even in a country known for its peaceful life, the violence encountered by the PECPs is frequent. The psychological impact of violence seems significant, even for so-called minor violence. This can lead to demotivation and fear at work, which must have a detrimental effect on medical care provided by PECPs. In order to reduce violence, it is important that comprehensive policies for the prevention of workplace violence are implemented within companies and institutions. For this, training in conflict management regarding abuse should be provided, but it is also important that victims find support from their employers. This study is the first step towards recognizing this problem of violence.

## Figures and Tables

**Table 1:** Types of workplace violence^*∗*^.

Verbal aggression: insults, offensive or condescending language, threats (any expression with the intention of hurting or denigrating).

Intimidation: being followed, menacing or frightening behavior (fist gestures, breaking things), harassment (any belittling, humiliating, annoying or irritating behavior).
Physical aggression: spitting, pushing, hitting, throwing objects with intention to hurt, kicking, punching, stabbing, etc.
Sexual harassment: sexual remarks or pleasantries, sexual gestures, demands for inappropriate sexual contact or exposure of genitals, breasts, buttocks, demands for social contact, phone numbers (exclusion of genital zone, buttocks, or breasts).
Sexual aggression: all nonconsensual acts including physical contact to genitalia, breasts, or buttocks.

^*∗*^Adapted from the definition of the Canadian Centre for Occupational Health and Safety [31].

**Table 2:** Demographic details (*n* = 273).

Sex, *n* (%)	Male	78 (28.6)
	Female	195 (71.4)

Age, *n* (%)	<35 years	119 (43.6)
	35–49 years	118 (43.2)
	>50 years	24 (8.8)
	Unknown	12 (4.4)

Profession, *n* (%)	Paramedic	157 (57.5)
	Ambulance technician	77 (28.2)
	Paramedic student	8 (2.9)
	Physician	31 (11.4)

Years of service, *n* (%)	0–2 years	41 (15.0)
	3–5 years	35 (12.8)
	6–10 years	52 (19.0)
	>10 years	145 (53.1)

Full/part-time, *n* (%)	Full 90–100%	214 (78.4)
	Part 50–89%	55 (20.1)
	<50%	4 (1.5)

Urban/rural, *n* (%)	>50% urban	74 (27.1)
	>50% rural	20 (7.3)
	Mixed	179 (65.6)

**Table 3:** Nature of acts of violence endured by PECPs during 2016^*∗*^.

	Total responses, *n* = 257	Males, *n* = 184	Females, *n* = 73
Verbal aggression, *n* (%)	255 (99.2)	182 (98.9)	73 (100)
Intimidation, *n* (%)	187 (72.8)	135 (73.4)	52 (71.2)
Physical aggression, *n* (%)	179 (69.6)	131 (71.2)	48 (65.8)
Sexual harassment, *n* (%)	42 (16.3)	17 (9.2)	25 (34.2)
Sexual aggression, *n* (%)	3 (1.2)	1 (0.5)	2 (2.7)

^*∗*^Cumulative results.

**Table 4:** Type of physical aggression^*∗*^.

	Total responses, *n* = 257	Males, *n* = 184	Females, *n* = 73
Spitting, *n* (%)	127 (49.4)	92 (50.0)	35 (47.9)
Pushing, *n* (%)	123 (47.9)	101 (54.9)	22 (30.1)
Kicking, *n* (%)	68 (26.5)	49 (26.6)	19 (26.0)
Punching, *n* (%)	59 (23.0)	51 (27.7)	8 (11.0)
Throwing objects intending to hurt, *n* (%)	45 (17.5)	33 (17.9)	12 (16.4)
Slap/smack, *n* (%)	39 (15.2)	27 (14.7)	12 (16.4)
Knifing, *n* (%)	17 (6.6)	14 (7.6)	3 (4.1)
Other^†^, *n* (%)	13 (5.1)	6 (3.3)	7 (9.6)

^*∗*^Cumulative results. ^†^Others = scratches, bites, punch on the throat, nondirected aggression, gripping, head butting, pulling the arm by force, and menaced or fired upon by a firearm.

**Table 5:** Psychological consequences of physical aggression.

Total responses	*n* = 179
Minor:	66 (36.9)
Mistrust, bitterness, anxiety, bad memories, disquiet, suspicion, smoking	

Moderate:	25 (14.0)
Hypervigilance, fear, hesitancy to enter certain areas, extreme sadness, rising insecurity at work	

Severe:	1 (0.6)
Depression, posttraumatic stress disorder, suicidal thoughts or actions	

**Table 6:** Reactions to violent patients (free replies)^*∗*^.

Total responses	*n* = 273
**1. Security measures**	
Seeking safety, *n* (%)	189 (69.2)
Call for help, *n* (%)	162 (59.3)
Protective measures, *n* (%)	29 (10.6)
Anticipation, *n* (%)	16 (5.9)
**2. De-escalating behavior**	65 (23.8)
**3. Restraining actions**	41 (15)

^*∗*^Cumulative results.

## Data Availability

The anonymized data that support the findings of this study are available from the corresponding author upon reasonable request.

## Abbreviations

PECPs: Prehospital emergency care providers
EMS: Emergency medical services.

## Supplementary Materials

Supplementary Materials This is the survey sent to all PECPs working in 2016 (416), containing seven questions on participants' demographic data and four sections with 30 questions detailing abuse encountered, its consequences, and changes in resulting practice for PECPs. Most questions are closed (yes/no answers) or have multiple answer choices. The final section has three open-ended questions. At the end, a free space allows comments.
