# Supplementary material for: Self-Reported Violence Experienced by Swiss Prehospital Emergency Care Providers
Source: Emerg Med Int. 2021 Dec 17;2021:9966950. doi: 10.1155/2021/9966950 (PMC8709758; doi:10.1155/2021/9966950)
Supplement: Supplementary Materials — This is the survey sent to all PECPs working in 2016 (416), containing seven questions on participants' demographic data and four sections with 30 questions detailing abuse encountered, its consequences, and changes in resulting practice for PECPs. Most questions are closed (yes/no answers) or have multiple answer choices. The final section has three open-ended questions. At the end, a free space allows comments. [file 9966950.f1.docx]

**Annex 1 :** Survey sent to participants of our study^[[1]](#footnote-2)^.

**Survey**

**« Violence towards prehospital emergency care providers »**

Present literature attests to an increase in violence towards prehospital emergency care providers (PECPs). However, there currently exists no documentation in Switzerland on this subject.

This survey is aimed at documenting the type of violence encountered by PECPs and evaluating its consequence.

Thank you for taking the time to reply top all the questions.

*Mandatory

1. **Demographic data**
2. Sex*

*One answer*

- Female
- Male

1. Year of birth*

For reasons of confidentiality, only give the YEAR of birth. We cannot accept full birthdates : simply put 01.01.yyyy. (ex : 01.01.1984)

1. Formation/function*

*One answer*

- Paramedic
- Ambulance technician
- Paramedic student
- Physician
- Other :

1. Years of service*

For students, tick « Other » and indicate the number of internships concluded

*One answer*

- 0-2 years
- 3-5 years
- 6-10 years
- > 10 years
- Other :

1. Full or part-time*

*One answer*

- Full time
- Part time

1. If part time, indicate the percentage of total worked

*Free response*

1. Urban or rural deployment*

*One answer*

- Principally urban (>50% missions in town)
- Principally rural (<50% missions out of town)
- Mixed

1. **Frequency of violence**

In this study, workplace violence is defined as follows :

*“any act at work during which a person is abused, menaced, intimidated, physically assaulted, sexual harassed or assaulted”* (Table 1)

Workplace violence includes :

**Verbal aggression :** insults, offensive or condescending language, threats (any expression with the intention of hurting or denigrating)

**Intimidation** : being followed, menacing or frightening **behaviour** (fist gestures, breaking things), harassment (any belittling, humilating, annoying or irritating **behaviour**)

Physical aggression : spitting, pushing, hitting, throwing objects with intention to hurt, kicking, punching, stabbing etc

**Sexual harassment** : sexual remarks or pleasantries, sexual gestures, demands for inappropriate sexual contact or exposure of genitals, breasts, buttocks, demands for social contact, phone numbers **(exclusion of genital zone, buttocks or breasts)**

**Sexual aggression** : All non-consensual acts including physical contact to genitalia, breasts or buttocks.

(Definition inspired by the Canadian Centre for Occupational Health and Safety, refined according to literature available in 2016).

1. Have you been the victim of violence at work ? *

*One answer per line*

|  | Yes | No |
| --- | --- | --- |
| During your career | ☐ | ☐ |
| During the year 2016 | ☐ | ☐ |

1. What was the nature of violence endured ? (according to the definitions below) *

*Several answers possible*

- Verbal aggression
- Intimidation
- Physical aggression
- Sexual harassment
- Sexual aggression
- I was never a victim of violence

1. Did you witness violence towards a colleague at work ? *

*One answer*

- Yes
- No

1. Did your colleagues report being victims of violence at work ? *

*.One answer*

- Yes
- No

1. During your career, how often have you endured violence at work ? *

If you have never endured violence, tick « Never » each time

*One answer*

|  | Never | 1-3 times/year | 1-3 times/year | 1 time/week | 2-3 times/week | Every day |
| --- | --- | --- | --- | --- | --- | --- |
| Verbal aggression | ☐ | ☐ | ☐ | ☐ | ☐ | ☐ |
| Intimidation | ☐ | ☐ | ☐ | ☐ | ☐ | ☐ |
| Physical aggression | ☐ | ☐ | ☐ | ☐ | ☐ | ☐ |
| Sexual harassment | ☐ | ☐ | ☐ | ☐ | ☐ | ☐ |
| Sexual aggression | ☐ | ☐ | ☐ | ☐ | ☐ | ☐ |

1. During 2016, how often have you endured violence at work ? *

If you have never endured violence, tick « Never » each time

*One answer per line*

|  | Never | 1-3 times/year | 1-3 times/year | 1 time/week | 2-3 times/week | Every day |
| --- | --- | --- | --- | --- | --- | --- |
| Verbal aggression | ☐ | ☐ | ☐ | ☐ | ☐ | ☐ |
| Intimidation | ☐ | ☐ | ☐ | ☐ | ☐ | ☐ |
| Physical aggression | ☐ | ☐ | ☐ | ☐ | ☐ | ☐ |
| Sexual harassment | ☐ | ☐ | ☐ | ☐ | ☐ | ☐ |
| Sexual aggression | ☐ | ☐ | ☐ | ☐ | ☐ | ☐ |

1. **Details of violence**
2. If you have endured verbal aggression at work, what sort was it ? *

If you have NOT been a victim of verbal aggression, tick « not relevant »

*Several answers possible*

- Insults
- Offensive or condescending language
- Shouts or cries with the intention of creating fear or offense
- Not relevant
- Other

1. If you have endured intimidation at work, what sort was it ? *

If you have NOT been a victim of intimidation, tick « not relevant »

*Several answers possible*

- Followed by the aggressor
- Menacing or frightening behaviour
- Intimidation at home or out of work context
- Not relevant

1. If you have endured physical aggression at work, what sort was it ? *

If you have NOT been a victim of physical aggression, tick « not relevant »

*Several answers possible*

- Pushing
- Slapping/smacking
- Spitting
- Throwing objects with intention of hurting
- Punching
- Kicking
- Knifing
- Not relevant
- Other

1. If you have endured sexual harassment at work, what sort was it ? *

If you have NOT been a victim of sexual harassment tick « not relevant »

*Several answers possible*

- Sexual pleasantries or humiliating sexual remarks
- Sexual or other offensive gestures
- Demand for sexual touching, & inappropriate handling of genitals, breasts or buttocks
- Insisting demands for meetings or telephone numbers
- Caresses, inappropriate physical contact out of the genital zone, buttocks or breast
- Not relevant
- Other

1. If you have endured sexual aggression at work, what sort was it ? *

If you have NOT been a victim of sexual aggression, tick « not relevant »

*Several answers possible*

- Actual or attempted non-consensual sexual contact
- Caresses, inappropriate physical contact on genital zone, buttocks or breast
- Not relevant
- Other

1. At what time were you a victim of work violence ? *

If you have NOT been a victim of work violence, tick « not relevant »

*Several answers possible*

- Morning (06:01 am -12:00 am)
- Afternoon (12:01 pm - 06:00 pm)
- Evening (06:01 pm - 12:00 pm)
- Night (00:01 am -06:00 am)
- Nit relevant

1. Where were you a victim of work violence ? *

If you have NOT been a victim of work violence, tick « not relevant »

*Several answers possible*

- At rescue site – public place
- At rescue site – home of patient or friend
- In the ambulance
- At hospital
- Out of work context
- Not relevant
- Other:

1. Who were the culprits of violence ? *

If you have NOT been a victim of work violence, tick « not relevant »

*Several answers possible*

- The patient
- Someone around of the patient
- A fellow medical care worker
- Not relevant
- Other :

1. **Consequences of violence on health**
2. Do you think violence is part and parcel of pre-hospital rescue work ? *

*One answer*

- Yes
- No

1. Concerning the risk of violence : did it make you afraid ? *

*One answer*

|  | Never | Rarely  (<25% of cases) | Occasionally  (<50% of cases) | Often  (<75% of cases) | Always (>75% of cases) |
| --- | --- | --- | --- | --- | --- |
| To go to work? | ☐ | ☐ | ☐ | ☐ | ☐ |
| On site of intervention | ☐ | ☐ | ☐ | ☐ | ☐ |

**Physical consequences of violence**

These consequences are divided into 3 groups : physical, psychological & professional.

Each type may have different consequences. Measure the degree of seriousness on a grade 0 to 3 : 0 (none, 1 minor, 2 moderate, 3 severe) as follows :

MINOR consequences : small wound without functional problem, pain resolved by simple analgesia…

MODERATE consequences : transitory functional difficulty, limitations of movement, plasters, dressings….

SEVERE consequences : functional deficit, disfiguration, surgical treatment needed

1. If you have endured workplace violence, what were the physical consequences ? *

If you have never been a victim of workplace violence tick « not relevant »

*One answer per line*

|  | Not relevant | 0 : No consequence | 1 : Minor | 2 : Moderate | 3 : Severe |
| --- | --- | --- | --- | --- | --- |
| Physical aggression | ☐ | ☐ | ☐ | ☐ | ☐ |
| Sexual harassment | ☐ | ☐ | ☐ | ☐ | ☐ |
| Sexual aggression | ☐ | ☐ | ☐ | ☐ | ☐ |

**Psychological consequences of violence**

These consequences are divided into 3 groups : physical, psychological & professional.

Each type may have different consequences. Measure the degree of seriousness on a grade 0 to 3 : (0 none, 1 minor, 2 moderate, 3 severe) as follows :

MINOR consequences : Mistrust, bitterness, anxiety, bad memories, disquiet, suspicion, smoking

MODERATE consequences : Hypervigilance, fear, hesitancy to enter certain areas, extreme sadness, rising insecurity at work

SEVERE consequences : Depression, post-traumatic stress disorder, suicidal thoughts or actions.

1. If you have endured workplace violence, what were the psychological consequences ? *

If you have never been a victim of workplace violence tick « not relevant »

|  | Not relevant | 0 : No consequence | 1 : Minor | 2 : Moderate | 3 : Severe |
| --- | --- | --- | --- | --- | --- |
| Verbal aggression | ☐ | ☐ | ☐ | ☐ | ☐ |
| Intimidation | ☐ | ☐ | ☐ | ☐ | ☐ |
| Physical aggression | ☐ | ☐ | ☐ | ☐ | ☐ |
| Sexual harassment | ☐ | ☐ | ☐ | ☐ | ☐ |
| Sexual aggression | ☐ | ☐ | ☐ | ☐ | ☐ |

*One answer per line*

**Professional consequences of violence**

These consequences are divided into 3 groups : physical, psychological & professional.

Each type may have different consequences. Measure the degree of seriousness on a gradfe 0 to 3 : (0 none, 1 minor, 2 moderate, 3 severe) as follows :

MINOR consequences : leave from work as a result of aggression

MODERATE consequences : reduction of work hours as a result of aggression

SEVERE consequences : ceasing work or changing job.

1. If you have endured workplace violence, what were the professional consequences ? *

If you have never been a victim of workplace violence tick « not relevant »

*One answer per line*

|  | Not relevant | 0 : No consequence | 1 : Minor | 2 : Moderate | 3 : Severe |
| --- | --- | --- | --- | --- | --- |
| Verbal aggression | ☐ | ☐ | ☐ | ☐ | ☐ |
| Intimidation | ☐ | ☐ | ☐ | ☐ | ☐ |
| Physical aggression | ☐ | ☐ | ☐ | ☐ | ☐ |
| Sexual harassment | ☐ | ☐ | ☐ | ☐ | ☐ |
| Sexual aggression | ☐ | ☐ | ☐ | ☐ | ☐ |

1. **Practical implications of violence.**
2. During the year, have you changes your practice concerning workplace violence ? *

*One answer*

- Yes *Continue at question 28*
- No *Continue at question 29*

1. Can you say how ? (Calling the police, Refusing to visit certain areas…) *

*Free response.*

1. Regarding serious violence, did you feel yourself to be adequately protected and upheld by the forces of order ? *

*One answer*

- Yes
- No

1. During the year, have you changed your workplace equipment ? *

*One answer*

- Yes *Continue at question 31*
- No *Continue at question 32*

1. Please detail what you have added to your equipment ? *

*Free response.*

1. What measures do you take when a patient is violent ? *

Write « none » if you do not take any specific measures.

*Free response*

1. Are you trained to deal with violence ? *

*One answer*

- Yes *Continue to question 34*
- No *Continue to question 35*

1. Do you deem this adequate? *

*One answer*

- Yes
- No

*Continue to question 36*

1. Are you interested in such training ? *

*One answer*

- Yes
- No

1. Did you report episodes of violence ? *

*One answer per line*

|  | Never | Rarely  (<25% of cases) | Occasionally  (<50% of cases) | Often  (<75% of cases) | Always (>75% of cases) |
| --- | --- | --- | --- | --- | --- |
| To your colleagues | ☐ | ☐ | ☐ | ☐ | ☐ |
| To your superior | ☐ | ☐ | ☐ | ☐ | ☐ |
| To your administration | ☐ | ☐ | ☐ | ☐ | ☐ |
| To the police | ☐ | ☐ | ☐ | ☐ | ☐ |

1. If you have not reported all episodes of violence, what are the reasons ? *

*Several answers possible.*

- Procedure too complicated
- No pointaas nothing will change
- Fear of repercussions from superiors for reporting such
- The patient was not in his normal state so not really responsible.
- I report all episodes of violence
- Other

1. Would you like to express further thoughts on the subject if workplace violence ?

*Free response.*

1. This questionnaire was sent to participants in French. It was translated afterwards by a professional translator. [↑](#footnote-ref-2)
